# Black Veterans Experiences with and Recommendations for Improving Weight-Related Health Care: A Photovoice Study

**DOI:** 10.1007/s11606-024-08628-7

**Published:** 2024-03-04

**Authors:** Jessica Y. Breland, Lamont Tanksley Sr., Michelle A. Borowitz, Dakota Houseknecht, Na’imah Muhammad, Susan D. Raffa, Katherine D. Hoerster

**Affiliations:** 1 Center for Innovation to Implementation, VA Palo Alto Health Care System, Menlo Park, CA USA; 2 Mental Health Service, VA Puget Sound Healthcare System, Seattle Division, Seattle, WA USA; 3 Health Services Research and Development, VA Puget Sound Healthcare System, Seattle Division, Seattle, WA USA; 4 VA National Center for Health Promotion and Disease Prevention, Durham, NC USA; 5 Department of Psychiatry and Behavioral Sciences, Duke University School of Medicine, Durham, NC USA; 6 Department of Psychiatry and Behavioral Sciences, University of Washington, Seattle, WA USA

**Keywords:** veterans, Black, African American, weight management, photovoice

## Abstract

**Background:**

Non-Hispanic Black or African American (hereafter Black) veterans lose less weight than other users of the Veterans Health Administration’s (VHA) weight management program (MOVE!), despite higher enrollment.

**Objective:**

To understand factors that affect weight loss disparities between Black veterans and other veterans.

**Design:**

Qualitative study using Photovoice methods.

**Participants:**

Self-identified Black veterans in MOVE! across the USA (two women, seven men).

**Approach:**

We conducted six virtual Photovoice sessions with Black veterans. Session one provided orientation to the goal of understanding factors that might affect weight loss disparities. Participants chose missions related to weight management and VHA care, bringing photos or other media (e.g., poems) to discuss during remaining sessions. Facilitators/participants identified themes related to each session in real time. Between and after sessions, facilitators/investigators conducted rapid qualitative analysis of transcripts/audio to group similar themes, identify illustrative quotes/photos/other media, and prepare dissemination products (e.g., this manuscript). Participants provided feedback on the manuscript during an additional session.

**Key Results:**

Themes were identified across three categories: (1) Food in Our Lives and Health Care; (2) Body Image; and (3) Healthcare Bias and Discrimination. The emotional impact of food and the negative effects of bias and discrimination on health care quality and trust were especially salient. Participants provided recommendations for weight-related and general care. Notable recommendations included the need for VHA to hire and retain providers—especially Black providers—who understand and respect Black patients and are committed to delivering evidence-based, culturally sensitive care. In addition, weight management care should be tailored to individual patients’ diets and health beliefs and deemphasize body mass index.

**Conclusions:**

Photovoice resulted in concrete targets that could reduce health disparities. Institutions should consider Photovoice and similar approaches to build trust with and incorporate input from marginalized communities. This approach requires sustained commitment from leaders to engage stakeholders and implement solutions.

**Supplementary Information:**

The online version contains supplementary material available at 10.1007/s11606-024-08628-7.

## INTRODUCTION

The Veterans Health Administration (VHA) serves over six million primary care patients.^1,2^ Many of these patients have obesity, including 44% of its non-Hispanic Black or African American (hereafter Black) users ^1^. VHA offers the MOVE! Weight Management Program for Veterans (MOVE!). It is a free behavioral weight management program based on the Diabetes Prevention Program and typically includes 12–16 weeks of group treatment focused on diet and exercise. A review of the efficacy and effectiveness literature found that MOVE! use is associated with modest weight loss.^3^ Unfortunately, Black veterans lose less weight than other veterans in MOVE!,^4^ despite enrolling at similar or higher rates.^5,6^ Similar weight loss disparities are seen outside VHA.^7–9^

Weight-related disparities are often conceptualized at the individual level. However, interacting systems related to economic stability, education, law, health care, and the environment (i.e., structural racism) lead to health disparities.^10^ Examples include past and present racism within the US medical system that is related to lower quality care, mistrust, and avoidance of health services^10–12^ and unfair housing and economic policies that prevent people from living in healthful environments and accruing the wealth required for health in the USA.^13^ The stress caused by trying to manage health under these conditions also contributes to weight-related disparities.^10^ For Black women, interacting stigma related to gender, weight, and race increases stress and exacerbates these pressures.^14^ Recent research demonstrates that higher levels on a county-level structural racism measure were associated with higher body mass index (BMI), with increasing effect sizes for White women, Black men, and Black women, but lower BMI for White men.^15^

Community-driven solutions may provide a way to counteract these dynamic, harmful systems. Photovoice is a qualitative method used within community-based participatory research. Photovoice gathers people with shared experiences with the goal of collectively identifying challenges and solutions through conversations grounded in photos participants provide to reflect their lived experience.^16^ Photovoice participants typically advocate for community-driven solutions to decision-makers. Therefore, it is both a research methodology and a tool for collective empowerment, capacity building, and advocacy. As such, it is well suited to address inequities. We conducted a virtual Photovoice group among Black veterans newly enrolled in MOVE!. Drawing on the Visual Voices method,^17^ participants also shared other forms of media to ensure a range of representations meaningful to participants. The goals were to learn about Black veterans’ daily experiences during MOVE! participation and to identify ways to improve weight-related VHA care for Black veterans.

## METHODS

### Recruitment and Consent

This study was approved by VA Puget Sound Health Care System’s Institutional Review Board. Recruitment occurred from September to October 2022. We used national VHA administrative data to identify veteran patients with Black or African American race, body mass index ≥30 (using most recent height and weight taken within 3 months), a MOVE! visit within the next month (but none in prior 3 months), and no dementia diagnosis (*N* = 813). We sought participants just planning to start MOVE! to understand experiences while they used MOVE! as opposed to retrospectively. We randomly selected 100 of those veterans for recruitment. Prospective participants were sent encrypted emails and postal mailings that included an information sheet and opt-in/out instructions. Staff called potential participants who did not respond within 2 weeks, reaching 30 people (see Supplementary Appendix).

Interested veterans reviewed the study information sheet with staff by phone, and if still interested were screened for eligibility. In addition to the criteria above, eligibility required self-identification as Black or African American, being ≥18 years old, being a veteran patient of a VA medical facility, routine telephone access, English fluency, willingness to be audio-recorded, and adequate hearing. If a person was eligible and interested, staff obtained oral consent.

### Photovoice Sessions

The six sessions were held virtually (October 2022–February 2023) and lasted ~85 min. Participants met over several months to capture experiences during MOVE! participation. Sessions were led by two Black facilitators (a woman psychologist [MAB], and a veteran man peer support specialist [LT]), who participated in approximately 5 h of training led by the investigators (a Black woman [JYB] and a White woman [KDH]).

Each session had a structured guide based on prior work.^18,19^ Facilitators followed the guide, using common group facilitation strategies (e.g., open-ended questions, asking for clarification, summarizing what was heard). Session one included an orientation to the project purpose, and how to take photos or use other media to document experiences. To reduce participation barriers related to digital photography and ensure participants could draw on a range of representations of their experiences and needs, we incorporated the Visual Voices modality,^17^ allowing participants to share other media (e.g., poetry, music, drawing). Sessions two through six started with review of the prior session to ensure takeaways/themes were accurate. Participants then discussed the current “mission” and related photos/media. Finally, facilitators and participants chose a mission for the next week, as is standard in Photovoice. Oftentimes, the next mission was chosen based on a theme that emerged during discussion of the current mission. If there was indecision over the choice, facilitators would select the mission that most participants wanted (e.g., via a vote). Participants provided all photos/other media and chose all missions (Supplementary Appendix provides mission list). Sessions were audio-recorded and transcribed. Participants were paid $40 per attended session.

### Analysis

During each session, facilitators and participants discussed key takeaways from the current and prior session. Staff completed memos after sessions (for facilitators) or after listening to audio (for investigators) to collect information on key themes, answers to research questions, photos/media that were particularly impactful during the session, potential biases, and other notes. Facilitators and at least one investigator also discussed key points after each session to inform the next session’s facilitator guide. Before the final session, the investigators and facilitators used the memos to develop a slideshow with key themes, suggestions, and images. During session six, participants offered feedback on the slideshow, highlighting quotes or themes that resonated with them.

After the final Photovoice session, JYB completed a rapid qualitative analysis process.^20,21^ The process entailed reviewing all recordings, transcripts, and memos while taking notes to refine quotes, images, and text related to existing themes (e.g., body image, discrimination, recommendations to improve care) and to identify new themes or quotes. Then, she used those notes to update the slideshow. Facilitators and investigators met twice to review/amend the updated slideshow. Finally, in June 2023, all former participants were invited to give feedback on the updated slideshow, which was used as the basis for this manuscript. Five former participants provided feedback, for which they received $20.

## RESULTS

Participants were nine veterans who self-identified as Black (two women, seven men). Mean age was 58 years (range, 42–69). We identified themes, presented in bold text, across three categories: (1) Food in Our Lives and Health Care; (2) Body Image; and (3) Healthcare Bias and Discrimination. In addition, we identified weight-related recommendations for care and recommendations for general VHA care.

### Food in Our Lives and Health Care

#### Food can bring up strong emotions and memories that can make some foods difficult to give up

**Figure 1:**
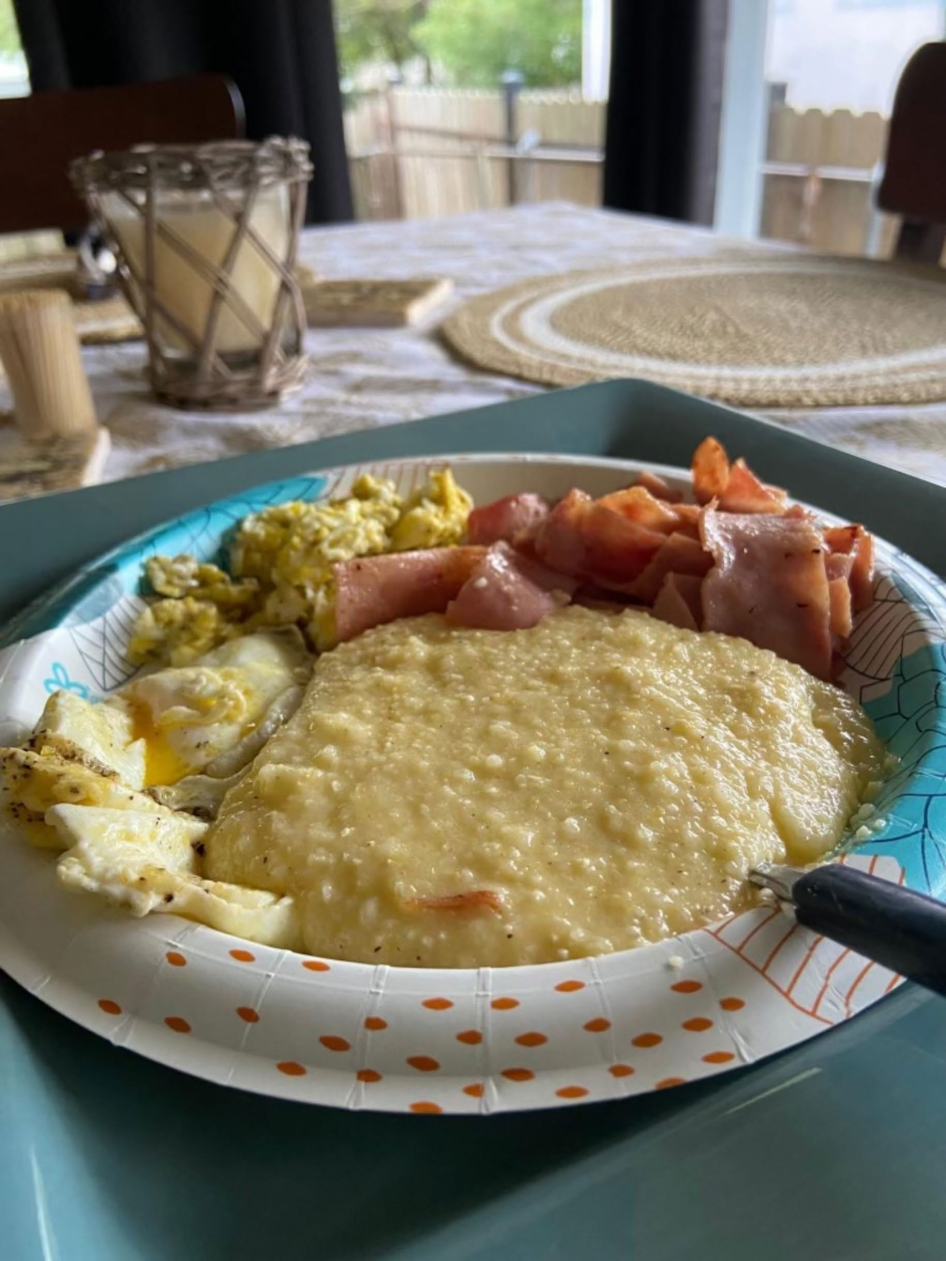
**A photo submitted by a participant during session 3.**

Throughout Photovoice sessions, participants described the joy eating can bring. This was especially pronounced when discussing foods with cultural or personal significance. An image that elicited strong, positive reactions from participants, facilitators, and investigators was of a bowl of grits with eggs and ham (Fig. 1). As a participant said, “When I first looked at [this picture]…my mother is from Louisiana, that was breakfast…rice with every meal, grits. It took me through [a] whole day [of my childhood] just looking at that one meal.” This participant captured how food is more than fuel — many foods taste great, while also providing meaningful connections to loved ones.

Relatedly, several participants described eating for emotional comfort. One participant described an image by saying, “I struggle with things that give me comfort or make me feel better, you know? And this is a prime example of stuff that makes me feel good, and makes me feel better. Fried chicken. Corn on the cob. Hot sauce. Salt, pepper.” His photo resonated with other group members who talked about eating similar (or different) foods to feel better. Given the strong feelings evoked by food, participants noted that changing dietary habits was difficult. One explained, “It’s easy for me to say that I want to eat healthier and exercise more, but actually implementing that into my lifestyle [is hard].” However, participants also noted that clinicians were unlikely to help change their eating habits without understanding the history and cultural relevance of foods commonly eaten in Black communities, for example, how a bowl of grits or plate of fried chicken represents more than just a collection of palatable calories (see next theme).

#### Doctors’ recommendations can take the joy out of eating

For some participants, difficulty in changing dietary habits stemmed from frustration with health care workers’ recommendations. This theme was illustrated by one participant:

You can’t eat too much beef because it will give you gout. You can’t eat pork because it will give you high blood pressure. You can’t eat fried foods because it’ll give you high cholesterol. So, what can I eat that I don’t have to worry about my health being involved in it? Why can’t I just eat something and enjoy it?

For others, it felt like MOVE! materials and dietary advice “vilified” foods they grew up eating. One person said, “… they had a list of words that were unhealthy. And it was fried, southern, smothered, alfredo…to my mind, well they’re just telling me everything that I like is bad.” Further, changes in recommendations and knowledge over time felt unfair. As a participant noted, “I feel like we ate plenty healthy … when we were growing up… And now I feel like… guess what? You’ve been tricked. None of this was healthy, none of this was good.” Participants described these experiences as especially frustrating given how enslavement and its aftermath shaped the diet of Black people in the USA, forcing them to make the most of limited options and incomes in ways that do not always conform to dietary advice, but as noted in the previous theme, are important and meaningful. Finally, there was a sense that clinicians did not understand how their recommendations negatively affected the experience of eating for Black veterans.

### Body Image, Self-image, and Health

#### Body image, self-image, and health are related and complex

Most of the discussion of this theme involved the interconnectedness of body image, self-image, and health. As a participant, who submitted an image of himself on a forklift, said, “Body image to me means health…You can tell when you start stepping on that forklift that you’re not doing right [with your health].” This quote also highlights how participants described using physical functioning to assess their health (see the next theme in this section for further detail).

#### Standards like BMI do not tell the whole story

When asked about BMI during session one, a participant said “I’m not a big fan of the BMI” because it was based on European standards. As a different participant said in a later session, “Doctors’ standards aren’t always the best standards…” In another session, a different woman described BMI as “insulting,” relating an illustrative experience:

I’ve never looked at myself as being someone overweight. I just say I’m a thick sister, I’m thick… But you go to the doctor and the doctor will tell you something entirely different ... I didn’t see myself as being somebody fat. …. It stinged a little bit but … ok, you’re seeing me as you’re seeing me, and I see me different... so maybe I can make some adjustments to the way I see me to better me.

This participant’s self-image did not match her doctor’s view. Yet, she was willing to acknowledge the doctor’s view, try to understand it, and use that understanding to improve her health. Participants felt that doctors and other clinicians did not give the same effort to understanding Black patients.

#### Many things beyond size (and including larger size) define beauty

Participants discussed an expansive view of beauty that included spirituality and generosity, in addition to physical attributes. Several participants noted that as they aged, physical beauty became less important to them, while noting they want to look “sharp.” Gender played a role, with men saying they appreciated larger women. And, at least one woman said she was treated differently and got less attention from men — including Black men — when she weighed more. A participant-submitted poem (Box 1) resonated strongly with the group. Participants agreed with the sentiment that there are general and weight-related biases against Black women. Notably, one participant asked for a copy of the poem to share with other women as encouragement.

**Box 1** A poem submitted by a participant

**Table:** 

**Big Black Girl by Jo Anne Hall**
They said she couldn’t be a woman Not with a shape like that Only the infantile and immature Could let her body get that Rep How dare she be, very disgustingly Walking around the midst of the public An embarrassment, shaming Come on, she got to be lonely Who would consider her lovely? Everyone knows she’s lazy. Let’s paper bag her non swag and give her time to someone else,… no lag The smaller the more golden, the fat not chosen
Number 1, The man should take her out only at night, ONLY he needs a real woman. You know: Gym, Business, Friends and close Family Rule Number 2, don’t tell her “I LOVE YOU” Next, don’t give her no respect The 4th thing is, is that she has to beg for the ring Fifth thing is that the fat lady can only sing….AND EAT
..when someone tells her that no one really likes her, they just tolerate her in order not to hurt her feelings, they don’t want to set blaze to her path in their dealings. That sex, food, warmth in the winter is the only doors men will enter. That her helping to pay for trips, whips and hair is the only reason her fake girlfriends act like they care. They get together and cause a stir, hiding from her.
They stay clear of her lair.
What if the Fat Girl wasn’t there?

### Health Care Discrimination and Bias

#### Black patients receive less attentive care than White patients, which leaves them feeling “unheard” and “rushed” and damages trust

All participants discussed the importance of feeling heard by providers and how being rushed made them feel unheard and not trust their clinicians or the health care system. They felt this was separate from the race and ethnicity of the provider.

Some participants described White veterans receiving better care or getting more time with providers: One said, “I have a buddy that’s a veteran, he’s Caucasian…if we go [to VA] at the same time, with the same ailment, the treatment is noticeably different.” Participants linked care experiences to individual provider differences, but also to racism and discrimination. Some, but not all, felt that non-Black providers did not understand Black culture and as a result they had difficulty connecting and building the trust required for medical care.

At the same, some participants had good experiences with VHA or did not feel racism and discrimination affected their care. One person said:

…For the most part, as far as my care is concerned, I definitely felt heard. And not only heard, but understood. So for the most part my experiences have been pretty positive. And again…I think we have a good cross section ethnicity wise.

Finally, several participants contextualized their experiences with discrimination and bias as being part of larger, interacting systems, including those related to housing and law. One participant’s comment about the interconnected nature of the food and medical systems connected many themes, “…all of the chemicals and all of these unhealthy things that are going into growing these products for us to eat brings about the health issues that we have, and to me it just makes the doctors make more money, and the pharmacist make more money. And we get sicker, and they get richer…” Other participants agreed with her, with one noting, “So the question becomes, are we defeating the purpose [of getting health care] by going to the healthcare provider…” The participants described both processed food and the stress of trying to find and afford organic food as negatively affecting health. Further, they did not view this as only an individual problem, instead relating it to a desire for different systems and for providers to acknowledge these systems and related difficulties.

#### Bias affects the ability of Black providers to get and stay in the medical field

During several sessions, participants talked about how racism and discrimination affected Black clinicians and VHA recruitment efforts. They also felt strongly that Black clinicians deserve equal pay and that VHA should focus on pay to attract Black staff. A participant noted — “I don’t believe [Black doctors are] going to stay with the VA if their salaries are not competitive.” This theme was often discussed in relation to the previously described lack of Black VHA clinicians and associated barriers to quality health care and trust. In addition to building trust, they noted that bringing in Black clinicians “…might bring some new insight to the health care system in general for the VA.”

### Recommendations to Improve Care

Participants described recommendations to improve VHA care for Black veterans across weight-related care and general health care. Regarding weight-related care, participants wanted more information about preparing healthful food, including information about how to make traditional foods healthier and how to incorporate preparation methods from other cultures. In addition, they wanted portion size recommendations and materials that did not vilify culturally important foods, and for providers to receive education to “understand [Black] culture and how we eat...Why I love grandma’s cooking, although there’s a lot of stuff that’s not good for me.” They also wanted providers to understand that a person’s size is not a direct reflection of their health, i.e., that BMI does not tell the whole story. Several participants described missing MOVE! sessions because they could not figure out how to attend virtual visits; therefore, ensuring virtual visit access is necessary.

Regarding recommendations to improve VHA health care in general, a common suggestion was for VHA to have more Black (as opposed to “diverse”) clinicians and staff. As a participant said, “[providers should] reflect the patients that they see.” Participants also talked about how their best providers, regardless of race or ethnicity, were those who spent time with them, communicating and listening. Related recommendations include that VHA dedicate resources to patient experience, including longer appointments to foster communication and trust. Some wanted more structured appointments (e.g., with information about visit length and lab results). As illustrated by the following quote, many participants also wanted providers to have education on how to respectfully care for Black veterans:

It’s usually frustrating to understand that your care is going to [be] based on a model that does not take into account any of your challenges. And you just have to make do and basically treat yourself. And until we get rid of that, and that trust, bond with the VA, I don’t know how it improves with the African American community.

Although this did not resonate with all participants.

The participants described learning a great deal from one another, including that some VHA facilities have many Black clinicians and staff. They all agreed that they wanted “to trust that there is consistency of care” for Black veterans across VHA facilities. Finally, they wanted Black veterans to have the opportunity to meet with other Black veterans and Black facilitators to have their voices heard. As one noted, “What’s important to me is that we’re not just another study that’s going to end up on a scholarly review page of papers of information, you know? That [doesn’t] invoke change.”

## DISCUSSION

This work used Photovoice methods to elicit information about drivers of weight loss disparities facing Black veterans in VHA’s MOVE! program. Findings suggest areas of future research and possible changes to clinical care and policy. For example, our study suggests that weight management programs may focus on foods common in Black communities when identifying “unhealthy” options, but not when suggesting healthy substitutions. As participants described, this can alienate patients by fostering concern about their diet without providing actionable information, an approach that decades of health psychology research suggests will not lead to behavior change.^22,23^ Perhaps instead of targeting types of food preparation in ways that may be biased against Black people, programs should use a “healthy plate” approach, focused on limiting portion size and fiber-rich vegetables, while providing examples consistent with culturally relevant food preparation.

Additionally, if proven effective, weight management treatments focused on stress management may reduce disparities.^24,25^ As in past qualitative work,^26^ several participants expressed that the stress imposed by getting medical care (e.g., excessive use of medications without proper counseling, shaming of foods with cultural significance) outweighed benefits they might get from medical care. The effects of stress are likely exacerbated by weight stigma, which is associated with lower quality care and negative health effects.^27^ Of course, stress management alone will be insufficient to address those concerns. Efforts to ameliorate the root causes of stress and disparities are needed. While not mentioned by name, some findings reflect the impact of structural racism (e.g., interacting social systems compounding the effects of inequity). This driver of inequity must be eliminated if true equity is to be realized.

Efforts to train, recruit, and retain high-quality Black health care workers should also be a top priority.^28^ This clear recommendation from participants is supported by recent findings that the presence of Black primary care providers is associated with lower mortality for Black people.^29^ Existing providers must respect the lived experiences of their patients and how those experiences inform behavior. As noted by the participants, this may require longer appointments. It could also include equipping veterans with tools like the AHRQ’s questions to ask of providers regarding prescribed medications.^30^ Providers and health care systems should continue to move away from using BMI as a measure of individual health status,^31^ and should adopt more body-accepting approaches to discussing weight with and providing weight management services to patients. Some providers may need additional training, although this recommendation is complicated by the lack of effective cultural competency training programs.^32^

### Limitations

Although we used proactive outreach to ensure participation in virtual sessions (e.g., reminder emails/texts, day-of calls, and troubleshooting technology challenges between sessions), not every participant attended each session (most attended two-thirds of sessions). However, there were ample opportunities for review and synthesis across sessions. In addition, results cannot generalize to all Black VHA patients, particularly due to our small sample with no participants from the Western USA, and because participation may have been motivated by strong judgments regarding care. Our study prioritized recruiting a smaller group of veterans to provide rich data over time and to build capacity for the group to advocate for improved health care over time. A single session model would have allowed us to obtain perspectives from more people across several focus groups but may have sacrificed the depth and complexity of connection and sharing among and by participants. Therefore, we believe the limitations are outweighed by strengths, such as the inclusion of women and men participants from across the USA, repeated input from participants to inform analysis, and visual/artistic representation to help tell veterans’ stories.

## Conclusions

Participants felt Photovoice sessions built trust with VHA, in part because facilitators were also Black. Representation is not sufficient to end disparities, but it can foster trust with marginalized groups. Photovoice sessions with Black veterans yielded actionable information to address inequities in weight management outcomes. A key finding was participants’ desire for care that accounts for individual needs of patients, while also being informed by knowledge of Black culture. This approach to care could reduce disparities in weight loss outcomes, while also addressing other disparities and building trust in VHA.

### Supplementary Information

Below is the link to the electronic supplementary material.

Supplementary file1 (DOCX 24 KB)
