# Supplementary material for: Black Veterans Experiences with and Recommendations for Improving Weight-Related Health Care: A Photovoice Study
Source: J Gen Intern Med. 2024 Mar 4;39(11):2033–40. doi: 10.1007/s11606-024-08628-7 (PMC11306895; doi:10.1007/s11606-024-08628-7)
Supplement: Supplementary file 1 — Supplementary file1 (DOCX 24 KB) [file 11606_2024_8628_MOESM1_ESM.docx]

**Supplementary Appendix**

**Table A.** *Photovoice recruitment status*

| **Status** | **N (%)** |
| --- | --- |
| Declined Screening | 16 (16%) |
| Enrolled (Attended Session) | 9 (9%) |
| Enrolled, but Dropped Out | 3 (3%) |
| Ineligible, Screen Fail | 1 (1%) |
| Lost to Follow-up | 1 (1%) |
| Not Called/Not Reached | 70 (70%) |

**Table B.** *Photovoice session topics (“missions”) and number of participants*

|  | **Mission** | **Number of Participants** |
| --- | --- | --- |
| Session 1 | Overview and goals of the project | 4 participants |
| Session 2 | Experiences with “kryptonite” foods | 6 participants |
| Session 3 | Body image | 6 participants |
| Session 4 | How can VA care be culturally, ethnically, and specifically suited to (better) care for Black veterans? | 7 participants |
| Session 5 | What we want our healthcare providers to know | 7 participants |
| Session 6 | How to obtain and retain African-American healthcare providers at VA | 8 participants |

Notes: 1) Missions for session 2-6 were chosen by participants. 2) Average number of sessions attended was four, with most (78%) attending at least two-thirds of sessions. 3) Introductory information covered during the first session (e.g., photography basics, mission information) was reviewed at subsequent sessions as needed.

**Table C.** *Characteristics of enrolled participants compared to those invited to participate*

|  | **Enrolled** | **Not Enrolled** |
| --- | --- | --- |
| **Age, Mean (SD)** | 58.1 (8.6) | 52.7 (12) |
| **Women, N (%)** | 2 (22.2%) | 37 (40.7) |
| **US Region, N (%)** |  |  |
| Midwest | 2 (22%) | 9 (10%) |
| Northeast | 1 (11%) | 9 (10%) |
| Southeast | 5 (56%) | 55 (60%) |
| Southwest | 1 (11%) | 8 (9%) |
| West | 0 (0%) | 10 (11%) |
